# Depichering the Effects of Astragaloside IV on AD-Like Phenotypes: A Systematic and Experimental Investigation

**DOI:** 10.1155/2021/1020614

**Published:** 2021-09-24

**Authors:** Xuncui Wang, Feng Gao, Wen Xu, Yin Cao, Jinghui Wang, Guoqi Zhu

**Affiliations:** ^1^Key Laboratory of Xin'an Medicine, Ministry of Education, Technology Center for Scientific Research, Anhui University of Chinese Medicine, Hefei 230038, China; ^2^Department of Neurology, The First Affiliated Hospital of University of Science and Technology of China, Anhui Provincial Hospital, Hefei 230001, China; ^3^School of Integrated Chinese and Western Medicine, Anhui University of Chinese Medicine, Hefei 230038, China

## Abstract

Astragaloside IV (AS-IV) is an active component in *Astragalus membranaceus* with the potential to treat neurodegenerative diseases, especially Alzheimer's diseases (ADs). However, its mechanisms are still not known. Herein, we aimed to explore the systematic pharmacological mechanism of AS-IV for treating AD. Drug prediction, network pharmacology, and functional bioinformatics analyses were conducted. Molecular docking was applied to validate reliability of the interactions and binding affinities between AS-IV and related targets. Finally, experimental verification was carried out in A*β*O infusion produced AD-like phenotypes to investigate the molecular mechanisms. We found that AS-IV works through a multitarget synergistic mechanism, including inflammation, nervous system, cell proliferation, apoptosis, pyroptosis, calcium ion, and steroid. AS-IV highly interacted with PPAR*γ*, caspase-1, GSK3*Β*, PSEN1, and TRPV1 after docking simulations. Meanwhile, PPAR*γ* interacts with caspase-1, GSK3*Β*, PSEN1, and TRPV1. *In vivo* experiments showed that A*β*O infusion produced AD-like phenotypes in mice, including impairment of fear memory, neuronal loss, tau hyperphosphorylation, neuroinflammation, and synaptic deficits in the hippocampus. Especially, the expression of PPAR*γ*, as well as BDNF, was also reduced in the hippocampus of AD-like mice. Conversely, AS-IV improved A*β*O infusion-induced memory impairment, inhibited neuronal loss and the phosphorylation of tau, and prevented the synaptic deficits. AS-IV prevented A*β*O infusion-induced reduction of PPAR*γ* and BDNF. Moreover, the inhibition of PPAR*γ* attenuated the effects of AS-IV on BDNF, neuroflammation, and pyroptosis in AD-like mice. Taken together, AS-IV could prevent AD-like phenotypes and reduce tau hyperphosphorylation, synaptic deficits, neuroinflammation, and pyroptosis, possibly via regulating PPAR*γ*.

## 1. Introduction

Alzheimer's disease (AD) is a neurodegenerative disease characterized by cognitive decline and behavioral impairment. The incidence of AD is increasing as the world population ages. According to the *World Alzheimer Report 2018* [1], there are more than 50 million people suffering from AD worldwide and it is predicted that by 2050, the number of AD patients will increase to 152 million. Currently, the pathogenesis and etiology of AD have not been fully elucidated, and there is no effective treatment for AD [2]. Remarkable efforts are made in developing strategies to resist mechanisms that lead to neuronal damage, synaptic deficits, neuroinflammation, and cognitive impairment [3–5]. Especially, amyloid-*β* (1-42) oligomers (A*β*O) accumulating in AD brains are linked to synaptic failure, neuroinflammation, and memory deficit [2, 6, 7].

Astragaloside IV (AS-IV), one of the major effective components purified from *Astragalus membranaceus*, has been documented in the treatment of diabetes and diabetic nephropathy [8, 9]. AS-IV has been reported to play a variety of beneficial roles in the prevention and treatment of neurodegenerative diseases with cognitive impairment [10]. Especially, AS-IV, as a selective natural PPAR*γ* agonist, inhibited BACE1 activity by increasing PPAR*γ* expression and subsequently reduced A*β* levels in APP/PS1 mice [11]. In addition, other studies pointed out that AS-IV could inhibit A*β*_1-42_-induced mitochondrial permeability transition pore opening, oxidative stress, and apoptosis [12, 13].

PPAR*γ* activation regulates the response of microglia to amyloid deposition, thereby increasing phagocytosis of A*β* and reducing cytokine release [14, 15]. In addition, PPAR*γ* agonists are able to improve the memory deficits in AD models [16, 17], which are further confirmed in clinical trials [18, 19]. In a previous study, we reported that AS-IV prevented A*β*O-induced hippocampal neuronal apoptosis, probably by promoting the PPAR*γ*/BDNF signaling pathway [20]. However, the findings were limited in the *in vitro* experiments, and systemic mechanisms have not been clearly disclosed.

In this study, we adopted a systematic study of the multiscale mechanism to investigate the treatment effect of AS-IV for AD, which combined the drug prediction, network pharmacology, functional bioinformatics analyses, and molecular docking. Subsequently, experiments were carried out to validate the potential mechanisms from the target of PPAR*γ*. This study would provide important implications for the treatment of AD.

## 2. Materials and Methods

### 2.1. Target Prediction

To obtain the molecular targets of AS-IV, a computer developed model SysDT based on random forest (RF) and support vector machine (SVM) algorithms [21], which integrates large-scale information on genomics, chemistry, and pharmacology was proposed to predict the potential targets with RF score ≥ 0.8 and SVM ≥ 0.7 as threshold. In addition, we also combined pharmacophore model [22] and structural similarity prediction methods to predict the targets of AS-IV [23].

### 2.2. Network Construction

**Figure 1:**
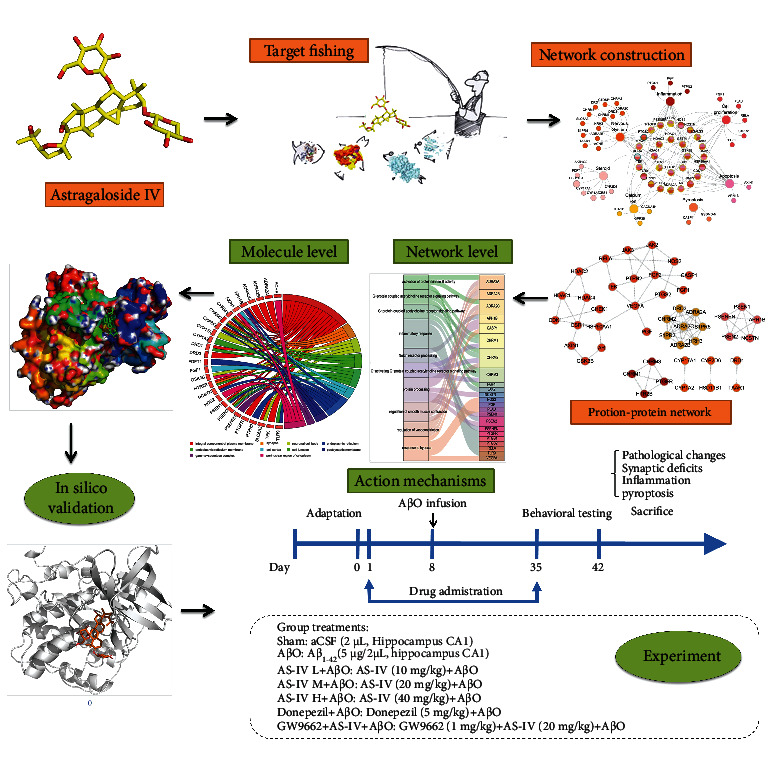
Experimental design. First, we screened the relevant targets via a comprehensive procedure; second, compound-target (C-T) and compound-target-function (C-T-F) were established to reveal the underlying molecular mechanisms; third, protein-protein interaction (PPI) network analysis and Gene Ontology (GO) enrichment analysis were performed to predict related targets; forth, we also studied the regulatory effect and specific mechanism of AS-IV on the AD via molecular docking and dynamics simulation; last, we investigated the effects of AS-IV on AD phenotypes in A*β*O-infused mice and further assessed the potential mechanisms. The mice were intragastrically administered daily with AS-IV (10, 20, and 40 mg/kg) for 7 days followed by intrahippocampal infusion of A*β*O or vehicle (aCSF) and then received AS-IV intragastrically or AS-IV plus GW9662 (1 mg/kg) intraperitoneally or donepezil (5 mg/kg) or the same volume saline intragastrically for 28 continuous days once per day. After that, the behavioral tests were conducted in the following one week, then the animals were sacrificed, and brain samples were collected. GW9662 (1 mg/kg) was coadministrated with 20 mg/kg of AS-IV or vehicle in the A*β*O-treated or aCSF-treated animals.

To visualize and analyze the relationship between the targets of AS-IV and their related biological functions, we screened the relevant function corresponding to the targets, introduced them into Cytoscape, and constructed the network. In this section, three networks including compound-target (C-T), compound-target-function (C-T-F), and protein-protein interaction (PPI) [24] were structured to unclose the multitarget and multifunction therapeutic effect of AS-IV in combating AD (Figure 1).

### 2.3. Gene Ontology (GO) Enrichment Analysis

Presently, to further investigate the vital biological process connected with the AS-IV-related targets, we mapped these targets to DAVID 1 for analyzing targets' biological meaning. The GO terms of biological process were utilized to symbolize genic function. Finally, those GO terms with *P* ≤ 0.05 and FDR ≤ 0.05 were selected in subsequent research.

### 2.4. Molecular Docking

To validate the C-T network, AS-IV was docked to its predicted targets (PPAR*γ*, caspase-1, GSK3*Β*, PSEN1, and TRPV1) by the AutoDock software version 4.1 package with default settings based on a powerful genetic algorithm method [25]. The X-ray crystal structures of targets (5GTN, 5IRX, 6IYC, 6PZP, and 6GN1) were taken from the RCSB Protein Data Bank. Each protein was prepared using methods such as adding polar hydrogens, partial charges, and defining the rotatable bonds. Finally, the results were analyzed in the AutoDock Tools.

### 2.5. Drugs and Reagents

*A*stragaloside IV (purity: HPLC > 98%), GW9662, and A*β*_1-42_ were purchased from Sigma-Aldrich. ELISA kits for A*β*_1-42_, IL-1*β*, IL-6, and TNF-*α* were obtained from Shanghai Jianglai Biotechnology. Antibodies against microtubule-associated protein tau (tau), p-tau, PPAR*γ*, postsynaptic density 95 (PSD95), synaptophysin (SYN), growth-associated protein 43 (GAP43), glial fibrillary acidic protein (GFAP), NOD-like receptor protein 3 (NLRP3), cleaved IL-1*β*, cleaved caspase-1, and GAPDH were obtained from Cell Signaling Technology. The antibody against activity-regulated cytoskeleton-associated protein (ARC) was obtained from Synaptic System. Antibodies against BDNF and microtubule-associated protein 2 (MAP-2) were obtained from Novus Biologicals. Alexa 488 or 594-labeled fluorescent secondary antibodies for immunofluorescence and 4,6-diamidino-2-phenylindole (DAPI) were obtained from Thermo Fisher Scientific. Prestained Protein Ladder was obtained from Thermo Fermentas. SuperSignal chemiluminescence reagents were obtained from Pierce.

### 2.6. Animals and Treatments

Male C57BL/6 mice (5-6 weeks old, 20-25 g) were obtained from the Beijing Weishang Lituo Technology Co., Ltd (SCXK (Beijing) 2016-0009). The mice were housed in groups of six per cage with controlled room temperature and humidity, under a 12 h light/dark cycle, with free access to food and water. The mice were adapted for one week before administration. All protocols were approved by the Animal Ethics Committee of Anhui University of Chinese Medicine (approval No. AHUCM-mouse-2019015), and the procedures involving animal research were in compliance with the Animal Ethics Procedures and Guidelines of the People's Republic of China.

The mice were randomly divided into the following groups (*N* = 8 per group) (Figure 1): a sham group, an A*β*O group, A*β*O plus AS-IV (10, 20, and 40 mg/kg/day, *i.g.*) groups, an A*β*O plus donepezil (5 mg/kg/day, *i.g.*) group, and an A*β*O plus AS-IV (20 mg/kg/day, *i.g.*) with GW9662 (1 mg/kg/day, *i.p.*) group. Drugs were administered once per day for one week followed by intrahippocampal infusion of A*β*O and continuously received AS-IV once per day for another four weeks. The dose of AS-IV and GW9662 was selected and modified based upon a previous study [11].

### 2.7. Preparation and Infusion of A*β*O

A*β*O were prepared from synthetic A*β*_1-42_ and incubated at 37°C for 1 week in a stock solution of 10 *μ*g/*μ*L, then routinely characterized by size-exclusion chromatography, as previously described [26, 27], and stored at -80°C until use after subpackaging. A*β*O were perfused at a final concentration of 2.5 *μ*g/*μ*L in aCSF.

For intrahippocampal infusion of A*β*O, mice were anesthetized with 5% isoflurane using a vaporizer system (RWD life Science Co., Ltd, Shenzhen, China) and maintained at 1% during the injection procedure, as previously described [26, 28]. A*β*O (5 *μ*g per site) were bilaterally delivered into the hippocampal CA1 region (stereotaxical coordinates relative to bregma: 2.3 mm anteroposterior, ±1.8 mm mediolateral, and 2.0 mm dorsoventral). Injections were performed in a volume of 2 *μ*L infused over 5 min, and the needle was left in place for 1 min to prevent backflow. Then, the mice were treated with penicillin to prevent infection. After the operation, the mice were kept under standard conditions with eating and drinking freely. Mice that showed signs of misplaced injections or any sign of hemorrhage were excluded from further analysis. Seven days before the A*β*O infusions, AS-IV (10, 20, and 40 mg/kg, once/day) was administered intragastrically in mice. Behavioral and pathological studies were performed 4 weeks postinjections of A*β*O.

### 2.8. Fear Conditioning

FC was evaluated as previously described [29]. On adaption day, mice were allowed to freely explore the conditioning chamber (UgoBasile, Gemonio, Italy) with a camera that was connected to the ANY-Maze™ software (Stoelting, NJ, USA, RRID:SCR_014289) for 5 min. On conditioning day, mice were placed into the same test chamber, and then, an 80 dB audiotone (conditioned stimulus: CS) was presented for 30 s with a coterminating 1.0 mA, 2 s long foot shock (unconditioned stimulus: US) three times at a 73 s interval. Then, mice were removed from the cage. The next day (contextual test), mice were put back into the conditioning chamber for 5 min, but without any audiotone or foot shock. On day 4 (cued test), the cover of the back and side chamber walls was removed. The mice were returned to the chamber followed by three CS (without a foot shock) that were presented for 30 s each. The freezing time was recorded for each test using the software.

### 2.9. Preparation of Hippocampal Tissue

Twenty-four hours after behavioral tests, some mice were anesthetized with 5% isoflurane and decapitated, and the hippocampi were then rapidly dissected on ice and snap-frozen in liquid nitrogen before storing at -80°C for biochemical tests. Others received transcardial perfusion with 4% paraformaldehyde (PFA), and then, the hippocampi were rapidly dissected and postfixed with 4% PFA overnight at 4°C followed by immersions in a solution containing 30% sucrose at 4°C for graded dehydration. Parts of the hippocampi were then cut into serial coronal frozen slices (20 *μ*m) for immunofluorescence assay, and other hippocampus samples were sliced into 4 *μ*m thick coronal slices for histopathological analysis.

### 2.10. Hematoxylin and Eosin (HE) Staining

After fixed in 4% paraformaldehyde for 24 h at room temperature, the hippocampal tissues were embedded in paraffin and coronally cut into 4 *μ*m thick slices (three slices per mouse). The tissues were dewaxed and successively rehydrated with alcohol (70%, 85%, 95%, and 100%), and then, the slices were stained with hematoxylin solution for 3 min followed by eosin solution for 2 min at room temperature. The slices were finally mounted by following dehydration with gradient alcohol and hyaline with xylenes and sealed with neutral gum. Representative photographs were captured by a light microscope with the DP70 software.

### 2.11. Enzyme-Linked Immunosorbent Assay

Hippocampal tissues were collected and homogenized with ice-colded saline, supplemented with protease and phosphatase inhibitor cocktails. The supernatants were collected for further analysis. The levels of endogenous A*β*_1-42_, IL-1*β*, IL-6, and TNF-*α* were determined using ELISA kits according to the manufacturer's instructions. The absorbance was recorded at 450 nm using a microplate reader (SpectraMax M2/M2e; Molecular Devices, Sunnyvale, CA, USA), and the concentrations of A*β*_1-42_, IL-1*β*, IL-6, and TNF-*α* were calculated from standard curves. Results were expressed as picograms per milliliter. Data were generated from 6-8 mice per group.

### 2.12. Immunofluorescence

Mice were sacrificed, and the hippocampi were snap-frozen in optimal cutting temperature (OCT) compound (Sakura Finetechnical, Japan). For immunofluorescence staining, the OCT-embedded hippocampi were cut into serial coronal 20 *μ*m thick slices and mounted on adhesive microscope slides. The slices were fixed with ice-colded acetone for 10 min and then blocked in 10% goat serum (containing 0.04% Triton X-100) for 90 min at room temperature. Subsequently, the slices were incubated with primary antibodies to MAP-2 (1 : 200), PSD95 (1 : 200), SYN (1 : 400), GAP43 (1 : 200), and GFAP (1 : 200) overnight at 4°C followed by incubation with Alexa-conjugated secondary antibodies (Thermo Fisher Scientific) for 2 h at room temperature. After counterstained with DAPI solution in the dark, the fluorescent images of slices were acquired using a confocal scanning microscope (FV1000, Olympus, Japan). At least six representative images were taken from each mouse for analysis by the Image J software (NIH, USA, RRID:SCR_003070).

### 2.13. Immunohistochemistry

Hippocampal slices were deparaffinized and rehydrated as described above. After antigen retrieval, slices were incubated with 3% H_2_O_2_ for 15 min and blocked in goat serum (containing 0.1% Triton X-100) for 30 min followed by incubation overnight at 4°C with primary antibodies to PPAR*γ* (1 : 200) and BDNF (1 : 200). Then, the slices were washed three times with PBS and incubated with the horseradish peroxidase (HRP) conjugated goat anti-rabbit or anti-mouse IgG (1 : 100) secondary antibody for 2 h at room temperature followed by incubation with 50 *μ*L 3,3′-diaminobenzidine (DAB) substrate (DAKO, Denmark) at room temperature for 10 min. The number of immunoreactive cells in the hippocampus was assessed using light microscopy (DP70; Olympus, Japan). At least three different fields (200 × 200 *μ*m) per slice were randomly selected for visualization. The mean optical density in the hippocampus region was calculated and used to determine PPAR*γ* and BDNF expression levels.

### 2.14. Golgi-Cox Staining

Golgi-Cox staining was performed to assess changes in dendrites and dendritic spines within hippocampal neurons using the FD Rapid GolgiStain™ Kit (FD NeuroTechnologies, USA) according to the manufacturer's instructions. Briefly, mice were anaesthetized with 5% isoflurane and decapitated, and the brains were rapidly removed and immersed in the impregnation solution (A : B = 1 : 1, total 2 mL/mouse) at room temperature in the dark and then replaced with new impregnation solution after 2 days. Two weeks later, brains were transferred into solution C and stored at 4°C for three days and then rinsed 3 times with PBST (containing 0.3% Triton X-100). Brains were then cut serially into 100 *μ*m coronal slices on a vibration microtome, and each slice was transferred to a gelatin-coated slide with solution C and then dried at room temperature at dark for up to 3 days. Then, the slices were placed in a mixture consisting of solution D, solution E, and distilled water (1 : 1 : 2) for 15 min followed by a dehydration series consisting of 50%, 70%, 85%, 95%, and 100% ethanol, for 3 applications at 5 min each. The slices were then transparented with xylenes and sealed with neutral gum for light microscopic observation. At least 3-5 dendritic segments of apical dendrites per neuron were randomly selected in each slice, and 5 pyramidal neurons were analyzed per mouse. For each group, the number of spines per dendritic segment of at least 3 mice was analyzed with using the Image J software (NIH, USA, RRID:SCR_003070). Results are expressed as the mean number of spines per 10 *μ*m.

### 2.15. Transmission Electron Microscopy

The hippocampi were rapidly dissected and placed in 2.5% glutaraldehyde at 4°C for 4 h followed by fixation with 1% osmium tetroxide for 1.5 h. After a series of gradient ethanol dehydrations, the tissues were immersed in propylene oxide for 30 min and then infiltrated with a mixture of propylene oxide and epoxy resin overnight. Then, the tissues were embedded in epoxy resin and placed in oven at 60°C for 48 h and then cut into serial ultrathin slices (70 nm thickness) and stained with 4% uranyl acetate for 20 min followed by 0.5% lead citrate for 5 min. The synaptic ultrastructures were observed under TEM (HT7700; Hitachi, Tokyo, Japan). In this study, at least 10 micrographs were randomly taken from each mouse and analysis of synaptic density was performed using the Image J software (NIH, USA, RRID:SCR_003070).

### 2.16. Immunoblotting

Hippocampi were collected and homogenized in RIPA buffer containing protease and phosphatase inhibitor cocktails, and the protein concentration was determined by bicinchoninic acid method (Pierce Biotechnology, Inc., USA). Then, 25 *μ*g total protein from each sample was resolved by 8-15% sodium dodecyl sulfate polyacrylamide gel electrophoresis at room temperature and electroblotted onto nitrocellulose membrane (GE Healthcare, USA) at 4°C for 2 h. Membranes were blocked with 5% nonfat milk dissolved in Tris buffered saline Tween (TBST) at room temperature for 2.5 h. Primary antibodies against PSD95 (1 : 1000), SYN (1 : 1000), GAP43 (1 : 1000), ARC (1 : 1000), PPAR*γ* (1 : 1000), GFAP (1 : 500), NLRP3 (1 : 1000), cleaved IL-1*β* (1 : 1000), cleaved caspase-1 (1 : 1000), and GAPDH (1 : 1000) were diluted in blocking solution and incubated with the membranes overnight at 4°C. After incubation with secondary anti-mouse or anti-rabbit IgGs (1 : 10000 in TBST) at room temperature for 90 min, membranes were washed in TBST buffer, developed with SuperSignal chemiluminescence substrate (Thermo Fisher Scientific, MA) and imaged with a chemiluminescence detector (FluorChem FC3; ProteinSimple, USA). The protein expression was quantified with the Quantity One software (Bio-Rad, Hercules, CA, USA, RRID:SCR_014280), and the densitometric plots of the results were normalized to the intensity of the GAPDH.

### 2.17. Statistical Analysis

All analyses were performed with the GraphPad Prism 5.0 software (GraphPad Prism, San Diego, CA, USA, RRID: SCR_002798), and data were expressed as mean ± standard deviation (SD). The statistical significance of difference between groups was evaluated using one-way ANOVA followed by Tukey test. *P* values of <0.05 were considered statistically significant.

## 3. Results

### 3.1. C-T Network

**Figure 2:**
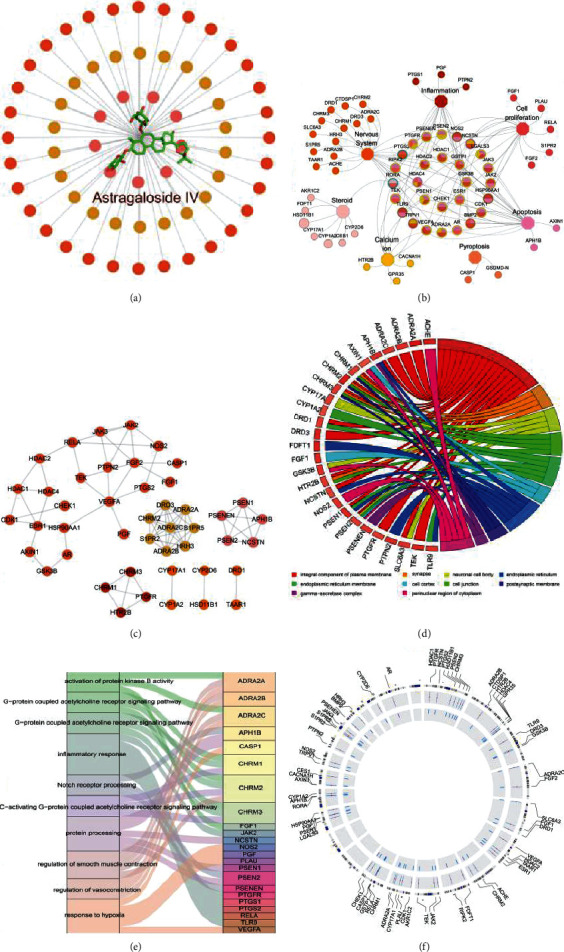
Work scheme of system pharmacology approach. (a) C-T network was constructed by linking the AS-IV with its potential targets (circles). (b) C-T-F network was constructed by the AS-IV and its function (octagons) and corresponding protein targets (circles). Among the targets, octagons with different colors represent the nervous system, inflammatory, cell proliferation, apoptosis, pyroptosis, calcium ion, and steroid targets, respectively. The circles in the middle are the nervous system, inflammatory, cell proliferation, apoptosis, calcium ion, and steroid overlapped targets. Node size is proportional to its degree. (c) The PPI network of AS-IV. The color and size of the node are proportional to the degree, and the color and thickness of the connecting line are proportional to betweenness centrality. (d) Gene Ontology analysis of AS-IV target genes. (e) Distribution of AS-IV target proteins in the underlying pathways involved in AD. (f) Distribution of AS-IV target proteins in chromosome.

In this study, we used a comprehensive method to screen AS-IV targets.  Figure 2(a) shows that there are 64 targets with the combining capacity to AS-IV. In this network, all these observations provide strong evidence that AS-IV works through a multitarget synergistic mechanism.

### 3.2. C-T-F Network

In order to further explain the pharmacological mechanisms of beneficial effects of AS-IV on AD, we classified the target functions of this compound and constructed the C-T-F network.  Figure 2(b) depicts the global view of the C-T-F network, in which the diamond, circle, and hexagon nodes represent AS-IV, targets, and the corresponding function of the targets, respectively. Further observation of this network shows that these 64 targets are related to 7 functions, including inflammation, nervous system, cell proliferation, apoptosis, pyroptosis, calcium ion, and steroid.

### 3.3. PPI Network

Proteins do not exert their functions independently of each other but interact together in the PPI network [30]. It is very helpful to understand the functions of proteins through analyzing the topological characteristics of proteins in PPI networks. Here, we constructed the PPI network of the 64 target proteins obtained from AS-IV and calculated the degree of each node. As shown in Figure 2(c), the degree of ADRA2A, ADRA2B, ADRA2C, CHRM2, S1PR5, S1PR2, DRD3, and HRH3 was the highest (degree = 7), followed by APH1B, PSENEN, PSEN1, PSEN2, and NCSTN (degree = 4), demonstrating that these proteins are hub targets and may be responsible for bridging other proteins in the PPI network.

### 3.4. GO Enrichment Analysis

Through the GO enrichment analysis (Figures 2(d)–2(f)), the targets were related to following biological processes, including G-protein coupled acetylcholine receptor signaling pathway (count = 2,3,6), protein kinase B activity (count = 1), Notch receptor processing (count = 5), protein processing (count = 7), and inflammatory response (count = 4). These processes were usually related to cell proliferation, gene transcription, differentiation, and development.

### 3.5. Molecular Docking

**Figure 3:**
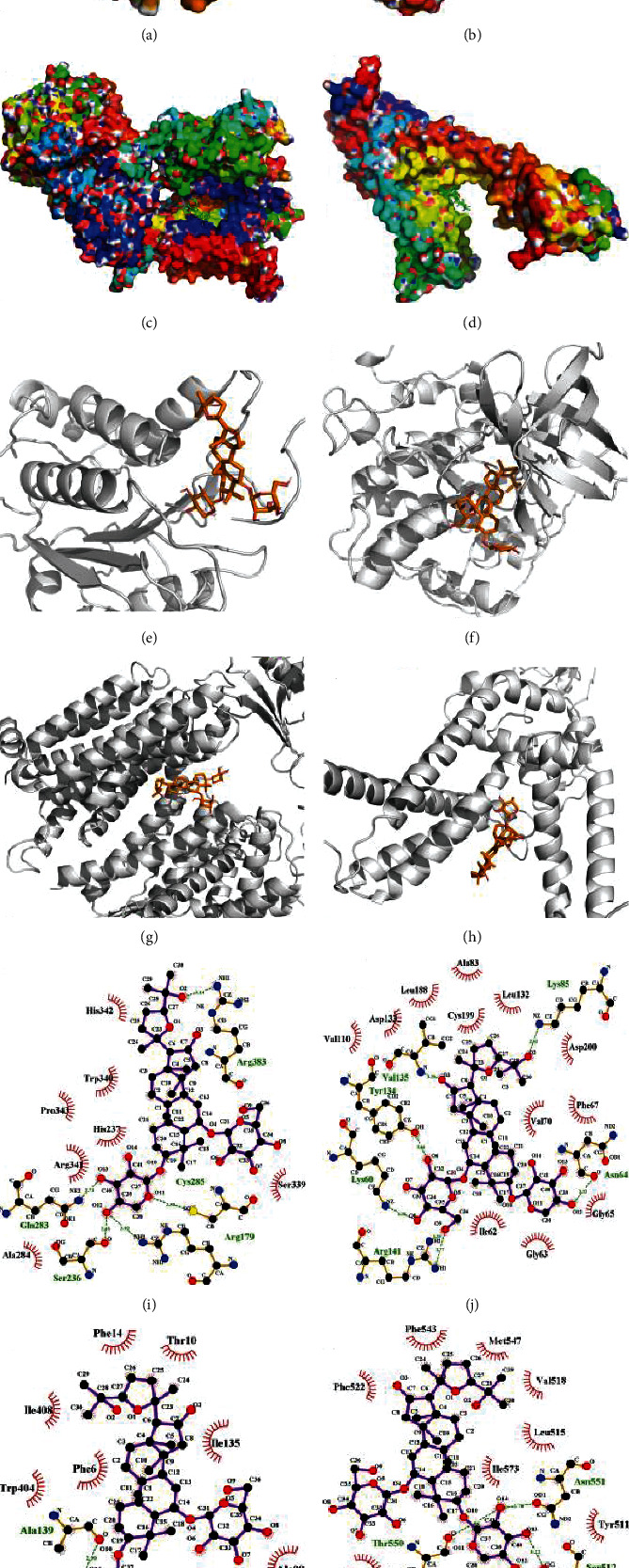
Binding conformations of AS-IV and four targets obtained from docking simulation. (a–d) The binding mode of AS-IV to (a) caspase-1, (b) GSK3*Β*, (c) PSEN1, and (d) TRPV1 in the active site. (e–h) Stereoview of binding mode for AS-IV with its receptors, i.e., (e) caspase-1, (f) GSK3*Β*, (g) PSEN1, and (h) TRPV1 in the binding site, where the H-bonds are depicted as the black dotted line. (i–l) The detailed view of the 2-D ligand interaction among AS-IV with (i) caspase-1, (j) GSK3*Β*, (k) PSEN1, and (l) TRPV1.

Figures 3(a)–3(l) depict the binding interactions of AS-IV with caspase-1, GSK3*Β*, PSEN1, and TRPV1 after docking simulations. The results showed that hydrophobic and H-bond interactions influenced the binding affinity of AS-IV to their target proteins (Figures 3(i)–3(l)). AS-IV was anchored into a hydrophobic pocket in caspase-1, GSK3*Β*, PSEN1, and TRPV1. In detail, for the binding pocket of caspase-1 with its ligand, there were large hydrophobic interactions formed by residues Trp340, Pro343, and Ala284; with respect to GSK3*Β*, the hydrophobic interactions were formed by residues Val110, Leu188, Ala83, Leu132, Val70, Phe67, and Ile62. Additionally, in PSEN1, it was formed by residues Phe14, Ile408, Ile135, Phe6, Trp404, Leu142, and Ala98. Also, in TRPV1, it was formed by residues Phe543, Phe522, Met547, Val518, Leu515, Ile573, Ala566, Leu553, and Ile569.

AS-IV interacted with many residues in the active sites of caspase-1, and three H-bond networks were formed (Figure 3). AS-IV forms H-bond networks with GSK3*Β* in Lys85, Val135, Lys60, Tyr134, Arg141, and Asn64. AS-IV forms H-bond interactions with PSEN1 in Ala139, while forms with TRPV1 in Asn551, Thr550, Arg557, and Ser512 (Figure 3). AS-IV is well suited to the receptor binding pocket as the binding of AS-IV to amino acids was tight and deep into the cavity. The binding free energy of AS-IV with caspase-1, GSK3*Β*, PSEN1, and TRPV1 was -5.30 Kcal/mol, -4.85 Kcal/mol, -6.41 Kcal/mol, and -6.07 Kcal/mol, respectively. These results indicated that AS-IV showed high binding affinities to its targets.

### 3.6. Interaction of PPAR*γ* with Caspase-1, GSK3*Β*, PSEN1, and TRPV1

**Figure 4:**
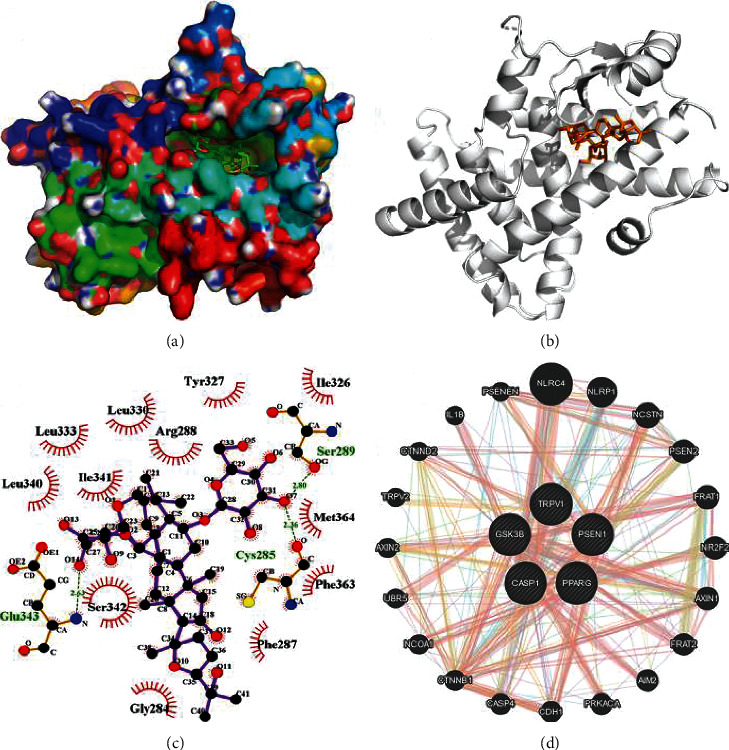
Interaction of PPAR*γ* with caspase-1, GSK3*Β*, PSEN1, and TRPV1. (a) The binding mode of AS-IV to PPAR*γ* in the active site. (b) Stereoview of binding mode for AS-IV with PPAR*γ* in the binding site, where the H-bonds are depicted as the black dotted line. (c) The detailed view of the 2-D ligand interaction among AS-IV with PPAR*γ*. (d) The PPI network of PPAR*γ* with caspase-1, GSK3*Β*, PSEN1, and TRPV1.

Figures 4(a)–4(c) depict the binding interactions of AS-IV with PPAR*γ* after docking simulations. For the target PPAR*γ*, AS-IV is directed toward the binding site and stabilized by the hydrogen-bonding interactions with Gln343, Cys285, and Ser289. Five critical proteins in the network, including PPAR*γ*, caspase-1, GSK3*Β*, PSEN1 and TRPV1, were selected to further validate the PPI. As shown in Figure 4(d), these five proteins showed a close interaction.

### 3.7. Effect of AS-IV on A*β*O-Induced Memory Impairment and Pathological Changes

**Figure 5:**
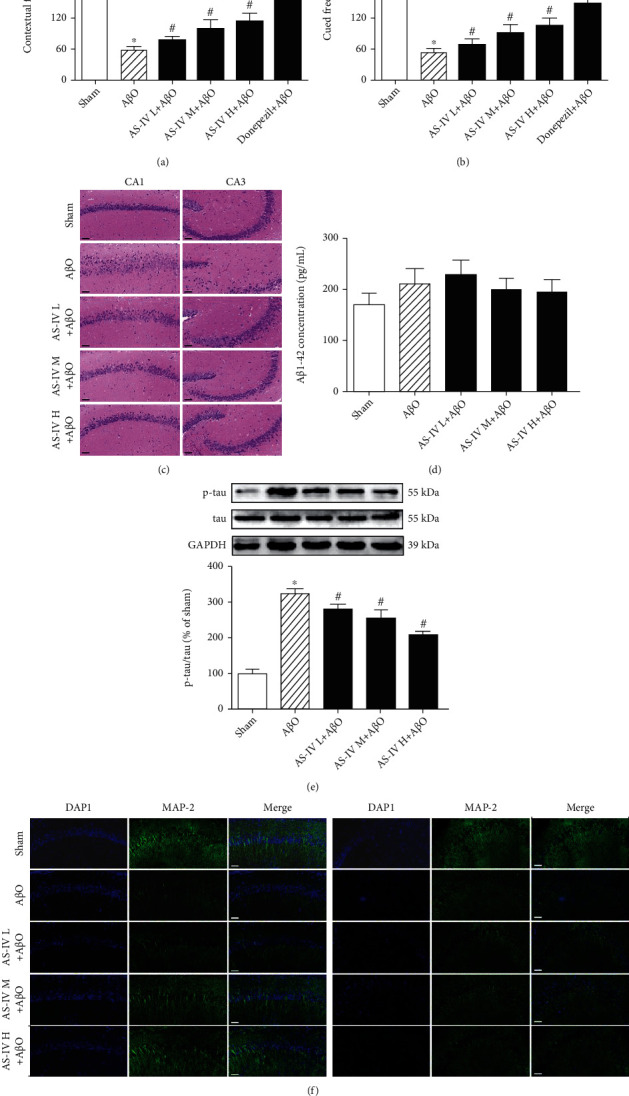
Effects of AS-IV on A*β*O-induced fear memory impairment and pathological changes in mice. (a) The freezing time of contextual memory. (b) The freezing time of cued memory. (c) Representative images of HE staining in the hippocampus (200x). Scale bar: 50 *μ*m. (d) The content of A*β*_1-42_ in the hippocampus measured by ELISA assay. (e) The expression of p-tau protein in the hippocampus measured by western blotting. (f) MAP-2 expression in the hippocampus measured by IF (×200). Scale bar: 50 *μ*m. Data are expressed as the mean ± SD (*N* = 8 or 6 per group). Compared with sham, ^∗^*P* < 0.05; compared with A*β*O, ^#^*P* < 0.05 (one-way ANOVA followed by the Tukey test).

FC task was further performed by the intensity of freezing to context and auditory cue to assess the effects of AS-IV on fear memory in A*β*O-infused mice. During the adaptation session, there was no difference in freezing time among experimental groups (data not shown). By exposure to the context and auditory cue, freezing response was both higher in sham mice than A*β*O-infused mice (Figures 5(a) and 5(b)). The freezing time was lower in A*β*O-infused mice after administration of AS-IV (10, 20, and 40 mg/kg) or donepezil, a positive control drug. These results suggested that AS-IV prevented A*β*O-induced contextual and cued fear memory impairments.

HE staining showed that the pyramidal cells in CA1 region of the hippocampus of sham mice had intact cell body and round nuclei with tight arrangement, and no cell loss was found. However, the pyramidal layer was disintegrated, and neuronal loss was observed in the CA1 region. Additionally, neurons with shrunken or irregular shape of cell bodies and degeneration of nuclei were also found in the hippocampus of A*β*O-infused mice (Figure 5(c)). It is worth mentioning that AS-IV (10, 20, and 40 mg/kg) administration attenuated the structural damage and loss of neurons to some extent relative to A*β*O-infused, which indicated a neuroprotective effect of AS-IV.

Next, the level of A*β*_1-42_ and phosphorylated tau expression was measured in the hippocampus. Results showed that there was no difference in the hippocampal A*β*_1-42_ level among experimental groups (Figure 5(d)). Compared with sham mice, the phosphorylated tau expression was increased significantly in A*β*O-infused mice. Compared with A*β*O-infused mice, AS-IV treatment reduced the hippocampal phosphorylated tau expression (Figure 5(e)).

We also observed MAP-2 expression in the hippocampus of mice by immunofluorescence assay. Results showed that there were a large number of MAP-2^+^ cells, with regular arrangement of neurons, obvious neurites arranged in bundles in the hippocampus of sham mice. Compared with sham mice, the numbers of MAP-2^+^ cells were remarkably reduced, the arrangement of dendrites was disordered, and the length of the neurites was significantly shortened in the hippocampus of A*β*O-infused mice. In contrast, AS-IV (20 mg/kg) administration reversed the inhibitory effects of A*β*O on the growth of MAP-2^+^ neurites (Figure 5(f)). Based on these findings, AS-IV administration alleviated A*β*O-induced neuronal injury and reduced tau phosphorylation in the hippocampus, but had no effect on endogenous A*β*_1-42_ level in A*β*O-infused mice.

### 3.8. AS-IV Suppresses A*β*O-Induced Synaptic Deficit in the Hippocampus

**Figure 6:**
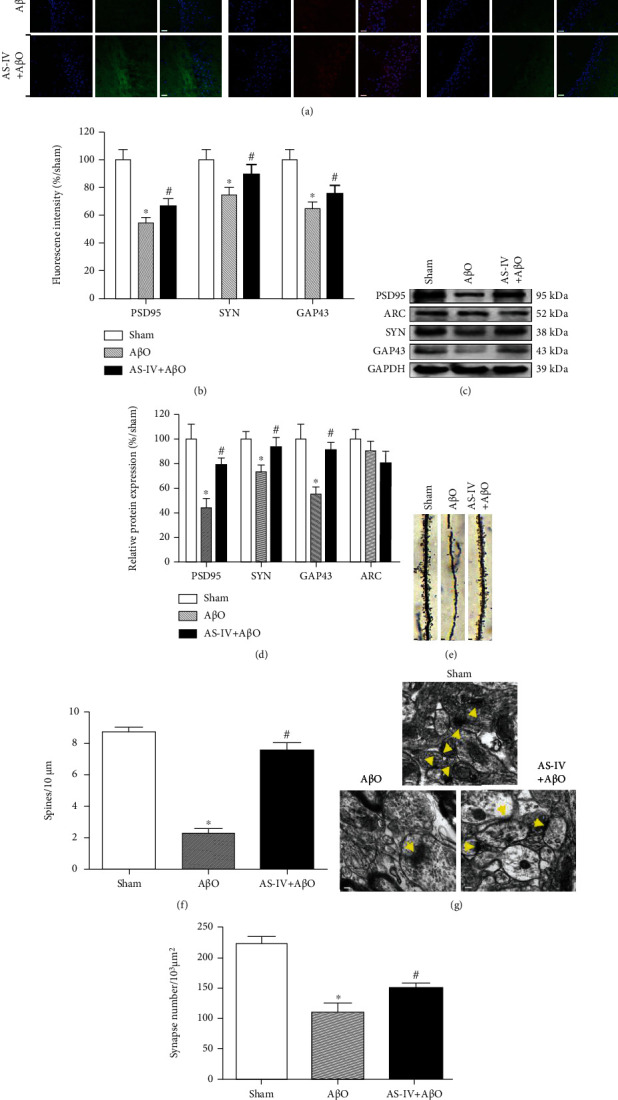
AS-IV suppresses A*β*O-induced synaptic deficits. (a) PSD95, SYN, and GAP43 expression in the hippocampus measured by immunofluorescence (×400). Scale bar: 5 *μ*m. (b) Fluorescence intensity data of PSD95, SYN, and GAP43 expression. (c) The protein expression of synaptic plasticity markers in the hippocampus measured by western blotting. (d) Relative quantitative data of PSD95, SYN, GAP43, and ARC protein expression. (e) Changes of dendritic spines in the hippocampus measured by Golgi-Cox staining (1000x). Scale bar: 2 *μ*m. (f) Quantitative data of dendritic spines in the hippocampus. (g) Ultrastructural changes of synapses in the hippocampus measured by TEM (8000x). Scale bar: 50 nm. (h) Quantitative data of synapses in the hippocampus. Data are expressed as the mean ± SD (*N* = 6 or 4 per group). Compared with sham, ^∗^*P* < 0.05; compared with A*β*O, ^#^*P* < 0.05 (one-way ANOVA followed by the Tukey test).

The effects of AS-IV on synaptic protein expression were investigated through determining the expression of PSD95, SYN, GAP43, and ARC. Results from immunofluorescence assays showed that the synaptic proteins PSD95, SYN, and GAP43 were all significantly reduced in hippocampal regions after A*β*O infusion when compared with sham mice. In contrast, AS-IV administration increased the immunoreactivity of PSD95, SYN, and GAP43 as compared to A*β*O-infused mice (Figures 6(a) and 6(b)).

The results from immunoblotting assays also showed that there was a significant decrease in the expression of PSD95, SYN, and GAP43 in response to A*β*O infusion, while AS-IV administration significantly ameliorated A*β*O-induced downregulation of these synaptic protein expressions in the hippocampus (Figures 6(c) and 6(d)). By contrast, there was no difference in these groups of mice regarding ARC expression (Figures 6(c) and 6(d)).

We next detected the density of dendritic spines in hippocampal neurons among experimental groups by Golgi-Cox staining assay. Results showed that the density of dendritic spines in hippocampal neurons of A*β*O-infused mice was significantly lower than that in sham mice, but these A*β*O infusion-induced changes in dendritic spine densities were significantly ameliorated by AS-IV (20 mg/kg) administration (Figures 6(e) and 6(f)).

We further used transmission electron microscopy to examine the synaptic ultrastructure of hippocampal neurons. Our data showed that A*β*O infusion resulted in a significant decrease of numbers of hippocampal synapses as compared to that of sham mice, whereas AS-IV (20 mg/kg) administration significantly ameliorated this synaptic loss (Figures 6(g) and 6(h)). Overall, the results indicate that AS-IV affords protection against A*β*O-induced synaptic deficits.

### 3.9. AS-IV Promotes A*β*O Infusion-Inhibited PPAR*γ* Expression in the Hippocampus

**Figure 7:**
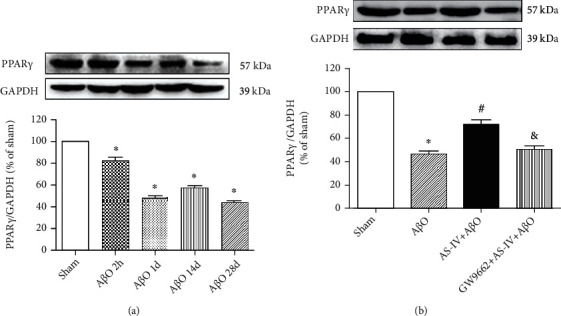
A*β*O infusion-inhibited PPAR*γ* expression was promoted by AS-IV. (a) The expression of PPAR*γ* protein at 2 h, 1 d, 14 d, and 28 d after A*β*O infusion in the hippocampus measured by western blotting. (b) The expression of PPAR*γ* protein after AS-IV administration in A*β*O-infused mice measured by western blotting. Data are expressed as the mean ± SD (*N* = 4 per group). Compared with sham, ^∗^*P* < 0.05; compared with A*β*O, ^#^*P* < 0.05; compared with AS-IV+A*β*O, ^&^*P* < 0.05 (one-way ANOVA followed by the Tukey test).

The hippocampus was collected at four time points after A*β*O infusion (2 h, 1 d, 14 d, and 28 d). The expression of PPAR*γ* significantly decreased at 2 h, 1 d, 14 d, and 28 d after A*β*O infusion (Figure 7(a)). By contrast, AS-IV attenuated the decrease of PPAR*γ* in A*β*O-infused mice. A specific PPAR*γ* antagonist, GW9662, was used to suppress PPAR*γ* activation in A*β*O-infused mice. Interestingly, the effect of AS-IV was blocked by GW9662 in the hippocampus of A*β*O-infused mice (Figure 7(b)).

### 3.10. AS-IV Inhibits A*β*O-Induced BDNF Reduction via Promoting PPAR*γ* Expression in Mouse Hippocampi

**Figure 8:**
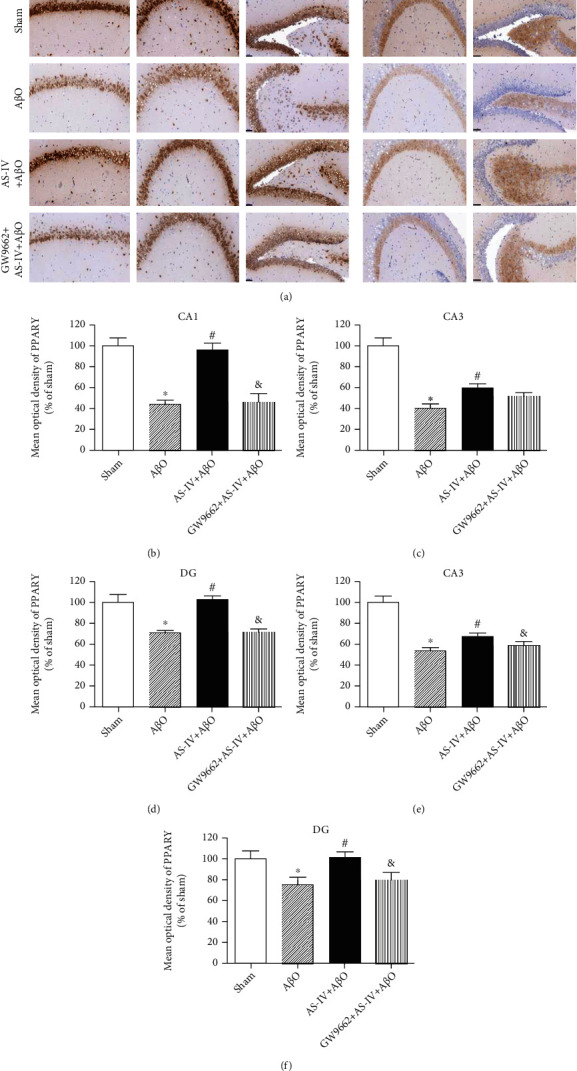
AS-IV inhibits A*β*O-induced BDNF reduction via promoting PPAR*γ* expression. (a) PPAR*γ* and BDNF expression measured by immunohistochemistry (×200). Scale bar: 50 *μ*m. (b–f) Quantitative data of PPAR*γ* and BDNF expression in different regions of the hippocampus. Data are expressed as the mean ± SD (*N* = 6 per group). Compared with sham, ^∗^*P* < 0.05; compared with A*β*O, ^#^*P* < 0.05; compared with AS-IV+A*β*O, ^&^*P* < 0.05 (one-way ANOVA followed by the Tukey test).

To further explore the underlying neuroprotective mechanism of AS-IV on A*β*O-infused mice, the levels of PPAR*γ* and BDNF in hippocampus were detected by immunohistochemistry. Compared with sham group, PPAR*γ* and BDNF immunoreactivity was decreased in the hippocampus of A*β*O-infused mice, whereas hippocampal immunoreactivity of PPAR*γ* and BDNF was higher in AS-IV-treated mice compared to A*β*O-infused mice (Figures 8(a)–8(f)). Additionally, the effect of AS-IV on the expression of BDNF and PPAR*γ* was blocked by GW9662 in the hippocampus of A*β*O-infused mice (Figures 8(a)–8(f)).

### 3.11. AS-IV Inhibits A*β*O-Induced Neuroinflammation via Promoting PPAR*γ* Expression

**Figure 9:**
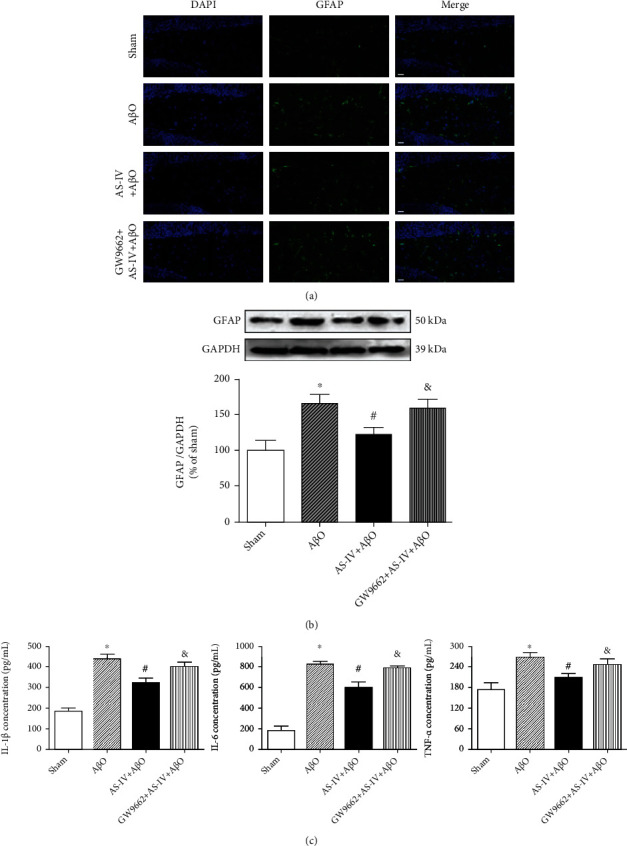
AS-IV inhibits A*β*O-induced neuroinflammation via promoting PPAR*γ* expression. (a) GFAP expression in the hippocampus measured by immunofluorescence (×400). Scale bar: 20 *μ*m. (b) The expression of GFAP protein in the hippocampus measured by western blotting. (c) The content of IL-1*β*, IL-6, and TNF-*α* in the hippocampus measured by ELISA. Data are expressed as the mean ± SD (*N* = 4 or 6 per group). Compared with sham, ^∗^*P* < 0.05; compared with A*β*O, ^#^*P* < 0.05; compared with AS-IV+A*β*O, ^&^*P* < 0.05 (one-way ANOVA followed by the Tukey test).

Our data showed that there were significant differences among the experimental groups with regard to the number of astroglia in DG region of the hippocampus, as detected by immunofluorescence (Figure 9(a)). Infusion of A*β*O induced a remarkable activation of astroglial responses in the hippocampus of mice, which was prevented by AS-IV (20 mg/kg) administration. Consistently, infusion of A*β*O also increased GFAP expression as determined with immunoblotting assay, while AS-IV (20 mg/kg) administration significantly suppressed GFAP expression in A*β*O-infused mice (Figure 9(b)). Furthermore, we asked whether PPAR*γ* mediated the beneficial effect of AS-IV on anti-inflammatory response in A*β*O-infused mice. Interestingly, PPAR*γ* inhibition by GW9662 blocked the inhibitory effects of AS-IV on GFAP immunoreactivity and expression in the hippocampus of A*β*O-infused mice (Figures 9(a) and 9(b)).

We measured the hippocampal IL-1*β*, IL-6, and TNF-*α* level in A*β*O-infused mice by ELISA. Results showed that A*β*O infusion led to an upregulation of IL-1*β*, IL-6, and TNF-*α* level in the hippocampus compared with sham mice, but AS-IV administration suppressed the upregulation of cytokines following A*β*O infusion. In line with the above findings, this effect of AS-IV was blocked by GW9662 (Figure 9(c)). These results suggest that AS-IV prevented the inflammatory response in the hippocampus via PPAR*γ*.

### 3.12. AS-IV Inhibits A*β*O-Induced Pyroptotic Cell Death via Promoting PPAR*γ* Expression

**Figure 10:**
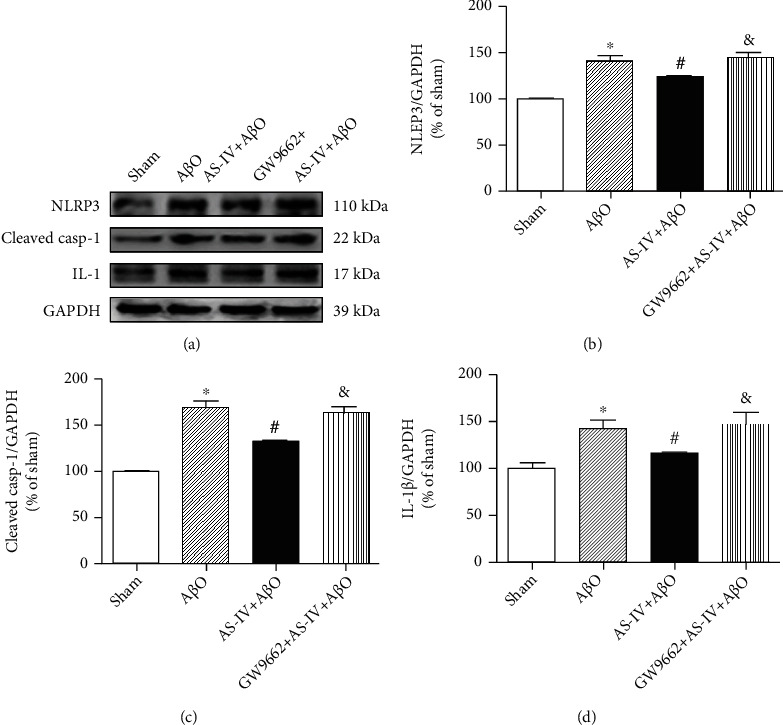
AS-IV inhibits A*β*O-induced pyroptotic cell death via promoting PPAR*γ* expression. (a) The protein expression of pyroptosis markers in the hippocampus measured by western blotting. (b) Relative quantitative data of NLRP3 protein expression. (c) Relative quantitative data of cleaved caspase-1 protein expression. (d) Relative quantitative data of IL-1*β* protein expression. Data are expressed as the mean ± SD (*N* = 4 per group). Compared with sham, ^∗^*P* < 0.05; compared with A*β*O, ^#^*P* < 0.05; compared with AS-IV+A*β*O, ^&^*P* < 0.05 (one-way ANOVA followed by the Tukey test).

As shown in Figures 10(a)–10(c), the protein expression of NLRP3 and cleaved caspase-1 was significantly elevated in the hippocampus of A*β*O-infused mice compared with sham mice. In contrast, AS-IV (20 mg/kg) administration suppressed A*β*O-induced expression of NLRP3, as well as cleaved caspase-1 in the hippocampus of A*β*O-infused mice.

As shown in Figures 10(a)–10(d), A*β*O infusion significantly increased the levels of IL-1*β* in the hippocampus, which was inhibited by AS-IV administration. In order to further confirm the role of PPAR*γ* in AS-IV-mediated suppression of A*β*O-induced pyroptosis, specific PPAR*γ* antagonist, GW9662, was used to suppress PPAR*γ* activation in A*β*O-infused mice. Interestingly, the effects of AS-IV against A*β*O-induced expression of NLRP3 and cleaved caspase-1 were blocked by GW9662. Moreover, the blockade of PPAR*γ* was able to significantly reverse the effect of AS-IV on A*β*O-induced proinflammatory cytokine IL-1*β* overexpression (Figures 10(a)–10(d)).

## 4. Discussion

In this study, we applied systemic pharmacology strategies and *in vivo* experiments to probe the mechanism of AS-IV in treatment of AD. AS-IV could interact with 64 targets, and those targets had multipharmacological properties relevant with nervous system, inflammation, cell proliferation, apoptosis, pyroptosis, calcium dysregulation, and steroid. Molecular docking suggested that AS-IV could regulate the AD-like phenotypes by binding with caspase-1, GSK3*Β*, PSEN1, and TRPV1. Furthermore, *in vivo* experiments evidenced that AS-IV promoted the expression of PPAR*γ* and BDNF in hippocampal neurons of mice infused with A*β*O and prevented synaptic deficits, inflammation, and memory impairments in AD-like mice. Consistent with the bioinformatics data, *in vivo* data also verified that AS-IV could suppress A*β*O infusion-induced neuronal pyroptosis. This systematic analysis provides new implications for the therapeutic of AD by AS-IV.

### 4.1. AS-IV Prevents AD Phenotypes through Multiple Mechanisms

In the present study, we screened 64 related targets of AS-IV and these targets together play important roles in the pathogenesis of AD, possibly through regulating cell proliferation, calcium dysregulation, inflammation, pyroptosis, and apoptosis [20, 31–33]. Specifically, the G-protein coupled acetylcholine receptor signaling pathway and protein kinase B/GSK3B axis are involved in the processes of AD pathogenesis, resulting in cognitive dysfunction [34–36]. Besides, the decrease of response to hypoxia and dysregulation of vasoconstriction could effectively ameliorate vascular dementia [37, 38]. Furthermore, the neuroinflammation caused by the generation of caspase-1-mediated IL-1*β* and IL-18 is involved in the development and progression of AD [32]. GSK3*Β* plays an important role in hyperphosphorylation of tau, which is one of the pathological features in AD [35]. PSEN1 mutation is a risk factor for AD [39]. Additionally, TRPV1, a nonselective cation channel, is involved in synaptic plasticity and memory [40]. Our molecular docking results demonstrate that AS-IV could integrate with caspase-1, GSK3*Β*, PSEN1, and TRPV1. The binding affinity of AS-IV is mainly through electrostatic, H-bond, and hydrophobic interaction, suggesting the reliability of the docking model. Therefore, AS-IV may improve cognitive impairment by binding to AD-related gene, such as caspase-1, TRPV1, PSEN1, and GSK3*Β*, reduce cell death, and ultimately inhibit AD-phenotypes.

### 4.2. AS-IV Reduces Tau Hyperphosphorylation in AD Model

A*β*O accumulate in the brains of AD patients and induce AD-like cognitive dysfunction [41]. Therefore, A*β*O-induced AD-like phenotypes may be a promising model to find treatments [41, 42]. In this study, we investigated the impact of A*β*O in the brains of mice and further confirmed the effect of AS-IV on memory formation in mice infused with A*β*O and to assess the mechanisms. Our results demonstrated that intrahippocampal infusion of A*β*O impaired both contextual and cued fear memory, which is consistent with previous study [43]. Conversely, AS-IV prevents A*β*O-induced contextual and cued fear memory impairment. Considering that hippocampus is an important brain region involved in the formation and expression of fear memory, our findings suggest that A*β*O infusion damaged the structure and function of hippocampus and subsequently blocked the formation of learning and memory, which can be prevented by AS-IV administration.

Similar to previous studies, our findings showed that A*β*O infusion induced neuronal loss, as well as increased tau phosphorylation, suggesting that the pathological changes of the hippocampus induced by A*β*O infusion may be the basis of AD-like behavioral changes [3, 44]. On the contrary, AS-IV inhibited the pathological changes of hippocampal neurons and tau phosphorylation induced by A*β*O infusion, which may contribute to memory improvement in AD-like mice. It is speculated that A*β* pathology in AD brain is earlier than those of tau, and neurofibrillary tangles develop downstream of toxicity induced by A*β* and eventually lead to neuronal death. Moreover, the mutual promotion between them accelerates the pathogenesis of AD, which is consistent with previous reports [44–46]. Certainly, we also note that A*β*O infusion has no effect on endogenous A*β*_1-42_ content in the hippocampus, suggesting it may not cause the increase and accumulation of A*β*, and formation of amyloid plaque in the brain. Through bioinformatics prediction, AS-IV could integrate with GSK3B tightly. As GSK3B is practically responsible for the hyperphosphorylation of tau, the tight interaction of AS-IV with GSK3B might contribute to the effects of AS-IV on the reduction of tau hyperphosphorylation.

### 4.3. AS-IV Prevents A*β*O-Induced Synaptic Deficit

Consistent with previous studies [47, 48], our findings demonstrated that A*β*O had neurotoxicity and synaptic toxicity before plaque formation in the brain, causing brain damage and eventually leading to AD-like behaviors. Given the mounting evidences that A*β*O caused synaptic deficits [3, 49, 50], elucidating the precise molecular pathways has important implications for treating and preventing the disease. Here, we demonstrate that A*β*O infusion reduced the immunoreactivity and expression levels of PSD95, GAP43, and SYN, which is similar to previous results [51, 52]. It has been shown that the SYN immune response density in the brain of transgenic mice is negatively correlated with A*β* levels, but has nothing to do with plaque loading, indicating that A*β* has synaptic toxicity when plaques are not formed [6]. We further found that AS-IV increased the immunoreactivity and expression levels of PSD95, GAP43, and SYN in the hippocampus of AD-like mice. PSD95, GAP43, and SYN are important markers of synaptic plasticity, and they are positively correlated with hippocampal learning and memory function [14, 53]. Furthermore, ARC plays a key role in synaptic plasticity and memory consolidation [54, 55]. Surprisingly, we note that there is no significant difference in ARC expression among the experimental mice, which suggested that A*β*O infusion did not target ARC. Our results of Golgi-Cox and TEM further showed that AS-IV increased the density of dendritic spines and synapse number in hippocampal neurons, which suggested that AS-IV improved synaptic structure damage and alleviated synaptic toxicity in the hippocampus of mice infused with A*β*O.

In a previous study, we reported that AS-IV promoted PPAR*γ* expression in cultured cells and activated the BDNF-TrkB signaling pathway [20]. Our *in vivo* findings further showed that PPAR*γ* expression in the hippocampus of mice infused with A*β*O was significantly decreased along with the reduction of BDNF expression, while AS-IV significantly prevented A*β*O-induced inhibition of PPAR*γ* and BDNF expression. Considering the important functions of BDNF-TrkB signaling pathway performed in synaptic function [29], those data further supported that AS-IV prevented A*β*O-induced synaptic deficit.

### 4.4. AS-IV Prevents A*β*O-Induced Neuroinflammation and Pyroptosis

Numerous studies have confirmed that neuroinflammation accelerates the pathogenesis of AD [46, 56, 57]. In this study, we found that A*β*O infusion increased the immunoreactivity and expression of GFAP and upregulated IL-1*β*, IL-6, and TNF-*α* levels in the hippocampus, which were reversed by AS-IV. These results suggested that AS-IV prohibited A*β*O-induced neuroinflammation in the brain, which was beneficial for cognitive function improvement, which further confirmed the network screening.

PPAR*γ* plays a neuroprotective role by reducing brain inflammation and A*β* production [58, 59]. Our findings showed that A*β*O infusion inhibited PPAR*γ* expression in mice, implicating that PPAR*γ* participated in inflammation response of AD-like mice. Furthermore, AS-IV blocked A*β*O-induced inhibition of PPAR*γ* expression. Pyroptosis is an inflammatory form of programmed cell death that has been reported in neurological pathogenesis [60]. Reducing pyroptosis was shown to alleviate cognitive impairment in AD animal models [61] and the progression of Parkinson's disease [62]. Interestingly, NLRP3 has been reported to initiate neuronal pyroptosis [63, 64]. Indeed, NLRP3 inhibition has been shown to exhibit neuroprotective effects through the suppression of pyroptosis [65] and improve neurological functions in a transgenic mouse model of AD [63]. In this study, we demonstrated that AS-IV could inhibit A*β*O-induced pyroptotic neuronal death, whereas PPAR*γ* antagonist GW9662 blocked the beneficial effect of AS-IV. In the systematic analyses, we also found that AS-IV had a high binding capacity with caspase-1, which might indicate the potential function of AS-IV in the pyroptosis.

### 4.5. AS-IV Reduces Tau Hyperphosphorylation, Synaptic Deficit, Neuroinflammation, and Pyroptosis via Regulating PPAR*γ*

In this study, we disclosed that A*β*O administration could progressively reduce PPAR*γ* expression in the hippocampus from 2 h to one day and kept the PPAR*γ* level at a relative low level from one day to 28 days. These data suggested that PPAR*γ* would be an initial event after A*β*O administration. AS-IV could prevent A*β*O-induced reduction of PPAR*γ*. The effects of AS-IV on brain inflammation, pyroptosis as well as synaptic deficit in A*β*O-induced AD phenotypes might be PPAR*γ*-dependent. On the one hand, PPAR*γ* antagonist blocked the effects of AS-IV on PPAR*γ* expression, brain inflammation, and pyroptosis as well as BDNF expression. On the other hand, PPI indicated that PPAR*γ*, caspase-1, GSK3*Β*, PSEN1, and TRPV1 had a close interaction. The reduced expression of PPAR*γ* induced by A*β*O administration contributes to deregulation of caspase-1, GSK3*Β*, PSEN1, and TRPV1, which might lead to brain inflammation and pyroptosis as well as synaptic deficit.

## 5. Conclusions

In summary, our present study indicates that AS-IV could suppress tau hyperphosphorylation, synaptic deficits, neuroinflammation, and pyroptosis to prevent AD-like phenotypes, likely through interactions of PPAR*γ* with caspase-1, GSK3*Β*, PSEN1, and TRPV1. This study offers a novel and reliable strategy for studying traditional Chinese medicine monomers.

## Data Availability

The data used to support the findings of this study are available from the corresponding authors upon request.

## Abbreviations

AD: Alzheimer's disease
AS-IV: Astragaloside IV
A*β*O: Amyloid-*β* (1-42) oligomers
PPAR*γ*: Peroxisome proliferator-activated receptor gamma
BDNF: Brain-derived neurotrophic factor
TRPV1: Transient receptor potential vanilloid 1
PSEN1: Presenilin 1
BACE1: *β*-Site amyloid precursor protein cleaving enzyme 1
GO: Gene Ontology
C-T: Compound-target
C-T-F: Compound-target-function
PPI: Protein-protein interaction
PSD95: Postsynaptic density 95
SYN: Synaptophysin
NLRP3: NOD-like receptor protein 3
GAP43: Growth-associated protein 43
GFAP: Glial fibrillary acidic protein
ARC: Activity-regulated cytoskeleton-associated protein
MAP-2: Microtubule-associated protein 2
DAPI: 4,6-Diamidino-2-phenylindole
aCSF: Artificial cerebrospinal fluid
FC: Fear conditioning
PFA: Paraformaldehyde
HE: Hematoxylin and eosin
ELISA: Enzyme-linked immunosorbent assay
IL: Interleukin
TNF-*α*: Tumour necrosis factor alpha
IHC: Immunohistochemistry
TEM: Transmission electron microscopy.
